# Meta-Analyses of 8 Polymorphisms Associated with the Risk of the Alzheimer’s Disease

**DOI:** 10.1371/journal.pone.0073129

**Published:** 2013-09-10

**Authors:** Xuting Xu, Yunliang Wang, Lingyan Wang, Qi Liao, Lan Chang, Leiting Xu, Yi Huang, Huadan Ye, Limin Xu, Cheng Chen, Xiaowei Shen, Fuqiang Zhang, Meng Ye, Qinwen Wang, Shiwei Duan

**Affiliations:** 1 Zhejiang Provincial Key Laboratory of Pathophysiology, School of Medicine, Ningbo University, Ningbo, Zhejiang, China; 2 The Neurology Department of the 148th Hospital of PLA, Zibo, Shandong, China; 3 Bank of Blood Products, Ningbo No. 2 Hospital, Ningbo, Zhejiang, China; 4 Ningbo Institute of Microcirculation and Henbane, Ningbo, Zhejiang, China; 5 The Affiliated Hospital, Ningbo University, Ningbo, Zhejiang, China; "Mario Negri" Institute for Pharmacological Research, Italy

## Abstract

**Aims:**

The aim of this study was to evaluate the combined contribution of 8 polymorphisms to the risk of Alzheimer's disease (AD).

**Methods:**

Through a comprehensive literature search for genetic variants involved in the AD association study, we harvested a total of 6 genes (8 polymorphisms) for the current meta-analyses. These genes consisted of *A2M* (5bp I/D and V1000I), *ABCA2* (rs908832), *CHAT* (1882G >A, 2384G >A), *COMT* (Val158Met), *HTR6* (267C >T) and *LPL* (Ser447Ter).

**Results:**

A total of 33 studies among 9,453 cases and 10,833 controls were retrieved for the meta-analyses of 8 genetic variants. It was showed that *A2M* V1000I (odd ratio (OR) = 1.26, 95% confidence interval (CI) = 1.07–1.49, P = 0.007), rs908832 allele of *ABCA2* (OR = 1.55, 95% CI = 1.12–2.16, P = 0.009), 2384G >A of *CHAT* (OR = 1.22, 95% CI = 1.00–1.49, P = 0.05) and Ser447Ter of *LPL* in the Northern-American population (OR = 0.56, 95% CI = 0.35–0.91, P = 0.02) were significantly associated with the risk of AD. No association was found between the rest of the 5 polymorphisms and the risk of AD.

**Conclusion:**

Our results showed that *A2M* V1000I polymorphism in German, Korean, Chinese, Spanish, Italian and Polish populations, rs90883 of *ABCA2* gene in French, American, Swiss, Greek and Japanese populations, 2384G >A of *CHAT* gene in British and Korean populations and *LPL* Ser447Ter in the Northern-American population were associated with the risk of AD.

## Introduction

Alzheimer's disease (AD) is the most common form of dementia among people over 65 years of age [1]. AD is predicted to affect 1 in 85 people globally by 2050 [1]. As an incurable degenerative disease, AD gets worse gradually and eventually leads to death. The features of AD development consist of loss of cognitive functions such as thinking, remembering, and reasoning, and ultimately leading to death. The averaged life expectancy after AD diagnosis is seven years [2]. Although hundreds of clinical trials have been conducted to find ways to treat the disease, none has claimed its effect of stopping or reversing the progressive symptoms. Because AD patients rely on others for assistance, it has imposed great economic costs on society [3], [4].

The cause for most AD cases is still largely unknown, although attempts have been made to explain AD by using the hypotheses based on acetylcholine [5], amyloid [6], [7], tau [7] and etc. In the present study, we performed meta-analyses for the variants on 6 protein encoding genes, including choline O-acetyltransferase (CHAT), catechol-O-methyltransferase (COMT), alpha-2-macroglobulin (A2M), 5-hydroxytryptamine receptor 6 (HTR6), ATP-binding cassette, sub-family A, member 2 (ABCA2), lipoprotein lipase (LPL). CHAT is an important enzyme catalyzing the biosynthesis of the neurotransmitter acetylcholine [8]. Altered protein levels of CHAT in neurons are shown to affect the symptoms of AD containing mild cognitive impairment [9], [10]. As one of the serotonin receptors, HTR6 plays a pivotal role in cognitive and memory processes [11] that are gradually damaged along with the AD progression. Involved in dopamine system, COMT is an important enzyme catalyzing the transfer of a methyl group from S-adenosylmethionine to catecholamines in the synapse [12]. *COMT* gene variant is associated with the volumes of ventral tegmental area where the gray matter correlates with cognitive and behavioral deficits in AD patients [13]. *A2M* encodes a protease inhibitor and cytokine transporter [14], [15]. A2M is important for the clearance and degradation of beta-amyloid [16], [17] which may lead to the pathogenesis of AD through the induction of tau phosphorylation [18]–[20]. *ABCA2* was cholesterol-responsive gene encoded a member of the superfamily of ATP-binding cassette (ABC) transporters [21]. Over-expression of *ABCA2* causes increased protein levels of amyloid beta precursor protein (APP) and beta-amyloid, both of which are important determinants of AD [22]. As a key enzyme to transfer fatty acids from triacylglyceride-rich lipoproteins, LPL is especially important in the process of cholesterol transport in neurons [23]. AD might be related to LPL protein that is one of component of amyloid plaques [24].

Associations between 8 single-nucleotide polymorphisms (SNPs) of the above 6 genes and AD have been reported in different ethnic groups [11], [25]–[57]. The results of these case-control studies for the above 8 genetic variants with AD differ across different groups. In the present study, we aimed to evaluate the combined contribution of the SNPs in these genes to AD susceptibility in different populations using a meta-analysis approach.

## Materials and Methods

### Literature search and study selection

Literatures were searched through the online databases from 1999 to 2012 using the following key words: “alzheimer's disease, association, SNP or polymorphism or variant or variation or mutation”. The involved databases include PubMed, Chinese National Knowledge infrastructure (CNKI), Embase, SpringerLink, and ScienceDirect. Reference lists in the harvested literatures were explored for additional case-control studies. The criteria for the selection of literatures in the meta-analyses were as followed: (1) the study was case-control association study; (2) allele or genotype information is available; (3) the involved genetic variants have not been studied in previous meta-analysis. The retrieved information consisted of the first author, the year of publication, the number of participants with the different allele (patients and healthy controls), and the odds ratios (ORs) values with 95% confidence intervals (CIs). In total, 33 publications addressing 8 polymorphisms were included in the current meta-analyses.

### Statistical Analysis

The meta-analyses were done using the Review Manager 5.0 software [58]. Total ORs with 95% CIs were estimated to evaluate the strength of the association between polymorphisms and AD risk. Heterogeneity was tested by the Cochran's Q statistic and I^2^ test [59]. A I^2^<50% denoted a non-signficant heterogeneity among the involved studies in the meta-analysis and fixed-effect model was used in the meta-analyses. The funnel plot was used to evaluate the publication bias in the meta-analysis. A two-sided P value <0.05 in the Z-test was treated as significant.

## Results

**Figure 1:**
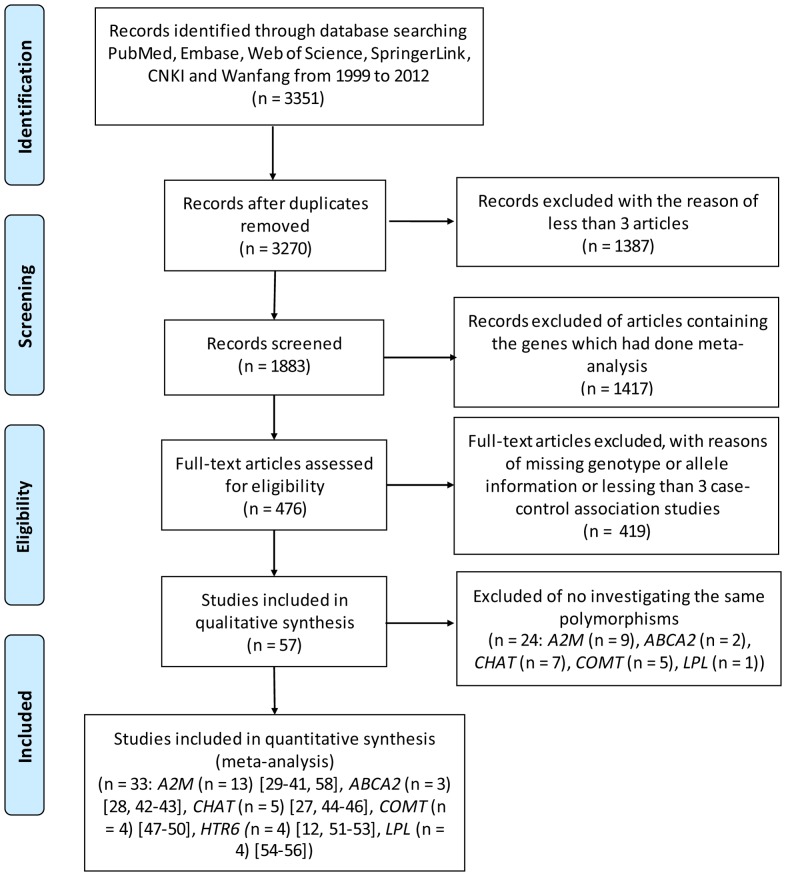
Flow diagram of the 8 meta-analyses.

As shown in Figure 1, our search for the case-control studies of AD retrieved 3,351 articles from PubMed, Embase, Web of Science, CNKI and Wanfang from 2000 to 2012. After removing the duplicated publications, we harvested 3270 articles. Among them, 1417 studies were discarded for their involvement in the previous meta-analyses. For the rest 476 studies, we filtered out a total of 428 articles because they failed to accumulate at least three independent genotypic datasets for the same genetic variants. At last, there were 33 case-control studies with 8 polymorphisms for the current meta-analyses (Figure 1).

**Figure 2:**
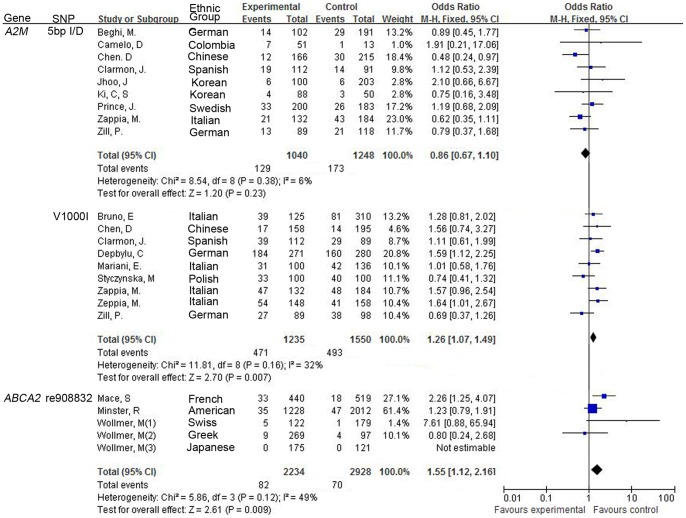
Forest plots for the relationship between SNPs (5bp I/D, V1001I, rs908832), and AD in the meta-analyses.

**Figure 3:**
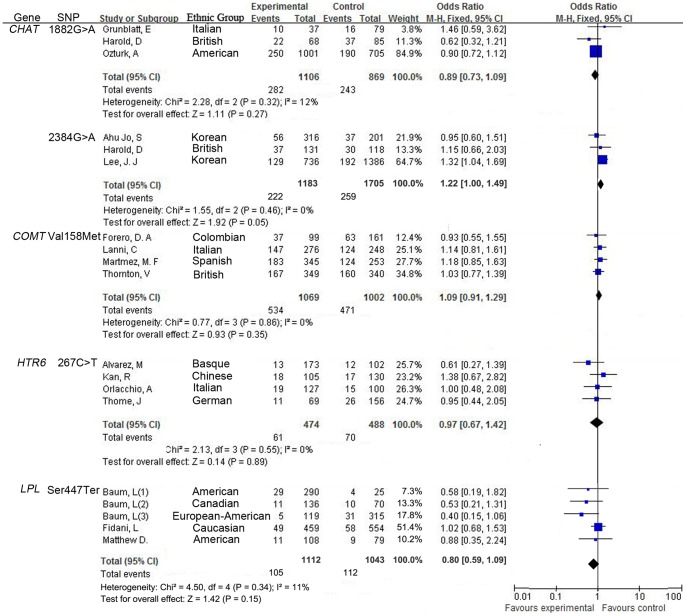
Forest plots for the relationship between SNPs (1882G >A, 2384G >A, Val158Met, 267C >T, rs2233678, rs2233679, Ser447Ter) and AD in the meta-analyses.

**Figure 4:**
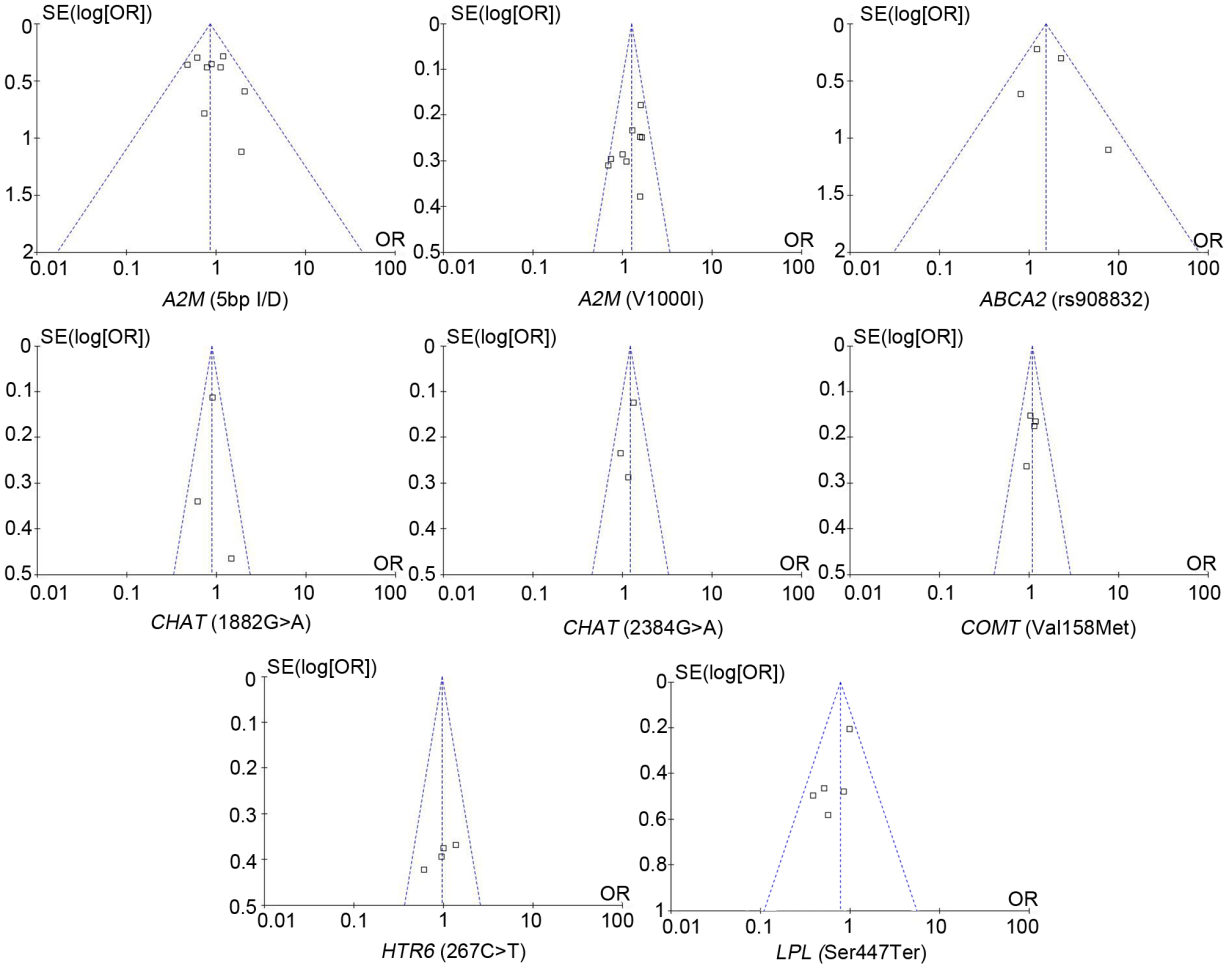
Funnel plots for the relationship between 8 SNPs and AD in the meta-analyses.

There was no evidence of statistical heterogeneity for all the SNPs in our meta-analysis. Minimal heterogeneity was observed for *A2M* 5bp-del (I^2^ = 4%, Figure 2), *CHAT* 1882G >A (I^2^ = 12%, Figure 3), *CHAT* 2384G >A (I^2^ = 0%, Figure 3), *COMT* Val158Met (I^2^ = 0%, Figure 3), *HTR6* 267C >T (I^2^ = 0%, Figure 3) and *LPL* Ser447Ter polymorphism (I^2^ = 11%, Figure 3). There was moderate heterogeneity for *A2M* V1000I (I^2^ = 32%, Figure 2) and *ABCA2* rs908832 polymorphism (I^2^ = 49%, Figure 2). As shown in the funnel plot, no obvious publication bias was observed for the 8 meta-analyses (Figure 4). The details were presented in the Tables 1 and 2.

**Table 1:** The characteristics of the enrolled SNPs (5bp I/D, V1001I, and rs908832).

Gene	SNP	Year	Author	Ethnic group	No. case/control	Genotype (case/control)			Allele (case/control)	
*A2M*	5bpI/D					I/I	I/D	D/D	I	D
		2000	Beghi, M	German	102/191	75/139	27/47	0/5	177/325	27/57
		2004	Camelo, D	Colombia	51/13	39/11	11/2	1/0	89/24	13/2
		2004	Chen. D	Chinese	166/215	150/175	8/20	8/20	308/370	24/60
		2003	Clarmon, J	Spanish	112/91	77/65	33/25	2/1	187/155	37/27
		2001	Jhoo, J	Korean	100/203	89/192	10/11	1/0	188/395	12/11
		2001	Ki, C, S	Korean	88/50	81/45	6/5	1/0	168/95	8/5
		2001	Prince, J	Swedish	200/183	139/137	56/40	5/6	334/314	66/52
		2002	Zappia, M	Italian	132/184	95/105	32/72	5/7	222/282	42/86
		2000	Zill, P	German	89/118	65/78	22/39	2/1	152/195	26/41
	V1000I					AA	AG	GG	A	G
		2010	Bruno, E	Italian	125/310	57/174	58/111	10/25	172/459	78/161
		2004	Chen, D	Chinese	158/195	127/168	29/26	2/1	283/362	33/28
		2003	Clarmon, J	Spanish	112/89	45/42	56/36	11/11	146/83	78/58
		2006	Depbylu, C	German	271/280	24/45	127/151	120/84	175/241	367/319
		2006	Mariani, E	Italian	100/136	49/70	41/49	10/17	139/189	61/83
		2003	Styczynska, M	Polish	100/100	49/40	37/41	14/19	135/121	65/79
		2002	Zappia, M	Italian	132/184	61/98	48/77	23/9	119/163	94/95
		2004	Zappia, M	Italian	148/158	65/85	58/65	25/8	188/235	108/81
		2000	Zill, P	German	89/98	44/32	37/56	8/10	125/120	53/76
*ABCA2*	rs908832					CC	CT	TT	C	T
		2005	Mace, S	French	440/519	376/484	63/35	1/0	815/1003	65/35
		2009	Minster, R	American	1228/2012	1160/1920	67/90	1/2	2387/3930	69/94
		2006	Wollmer, M	Swiss	122/179	113/177	9/2	0/0	235/356	9/2
				Greek	269/97	253/89	15/8	1/0	521/186	17/8
				Japanese	175/121	175/121	0/0	0/0	350/242	0/0

a:N.A. denotes not available.

**Table 2:** The characteristics of the enrolled SNPs (1882G >A, 2384G >A, Val158Met, 267C >T, and Ser447Ter)^a^.

Gene	SNP	Year	Author	Ethnic group	No. case/control	Genotype (case/control)			Allele (case/control)	
*CHAT*	1882G >A					GG	GA	AA	G	A
		2011	Grunblatt, E	Italian	37/79	24/51	7/26	6/2	55/128	19/32
		2003	Harold, D	British	68/85	34/49	25/33	9/3	93/131	43/73
		2006	Ozturk, A	American	1001/705	563/369	376/292	62/44	1502/1030	500/380
	2384G >A					GG	GA	AA	G	A
		2006	Ahu Jo, S	Korean	316/201	211/192	99/7	6/2	521/454	111/74
		2003	Harold, D	British	131/118	69/65	51/47	11/6	189/177	73/59
		2012	Lee, J. J	Korean	736/1386	505/1023	205/342	26/21	1215/2388	257/384

*COMT*	Val158Met					AA	AG	GG	A	G
		2006	Forero, D. A	Colombian	99/161	41/53	43/90	15/18	125/196	73/126
		2012	Lanni, C	Italian	276/248	61/57	141/131	74/60	259/248	293/248
		2009	Martmez, M. F	Spanish	345/253	74/67	176/125	95/61	324/259	366/247
		2011	Thornton, V	British	349/340	99/105	167/151	83/84	365/361	333/319
*HTR6*	267C >T					CC	CT	TT	C	T
		2003	Alvarez, M	Basque	173/102	149/81	23/18	1/3	321/180	25/24
		2004	Kan, R	Chinese	105/130	70/101	35/25	0/4	175/227	35/33
		2002	Orlacchio, A	Italian	127/100	92/76	32/19	3/5	216/171	38/29
		2001	Thome, J	German	69/156	50/107	17/47	2/2	117/261	21/51
*LPL*	Ser447Ter					Ser/Ser	Ser/Ter	Ter/Ter	Ser	Ter
		2000	Baum, L	American	290/25	N.A	N.A	N.A	522/43	58/7
				Canadian	136/70	N.A	N.A	N.A	250/121	22/19
		1999	Baum, L	European-American	119/315	N.A	N.A	N.A	229/568	9/62
		2002	Fidani, L	Caucasian	459/554	368/444	85/104	6/6	821/992	97/116
		2002	Matthew D	American	108/79	N.A	N.A	N.A	194/140	22/18

a: N.A. denotes not available.

Meta-analysis of A2M V1000I polymorphism involved 9 studies among 1235 cases and 1550 controls. As shown in Figure 2, V1000I was risk factors to AD onsets (OR = 1.26, 95% CI = 1.07–1.49, P = 0.007, Figure 2). A strong association between rs908832 of ABCA2 gene and AD was observed in the meta-analysis of 5 studies among 2234 cases and 2928 controls (OR = 1.55, 95% CI = 1.12–2.16, P = 0.009, Figure 2). Moderate association was found between the CHAT 2384G >A polymorphism and AD in the meta-analysis of 3 studies among 1183 cases and 1705 controls (OR = 1.22, 95% CI = 1.00–1.49, P = 0.05, Figure 3). As shown in Figure 3, no association of LPL Ser447Ter polymorphism with AD was found in the meta-analysis of 5 studies among 1112 cases and 1043 controls (OR = 0.8, 95% CI = 0.59–1.09, P = 0.16). However, the subgroup analysis by ethnicity found that LPL Ser447Ter polymorphism in the Northern-American population was associated with the risk of AD (OR = 0.56, 95% CI = 0.35–0.91, P = 0.02, Figure S1).

In order to test the robustness of the results in the meta-analyses, we perform a series of subgroup meta-analyses by excluding each study in turn, and the results showed that there was a significant association between the 2 SNPs (A2M V1001I, ABCA2 rs908832) with AD (P<0.05), except for the exclusion of Depbylu's study (A2M V1001I) and the Mace's study (ABCA2 rs908832) (Table S1). For LPL Ser447Ter, the subgroup meta-analysis by excluding Fidani's study found a significant association between LPL Ser447Ter and the risk of AD (Z = 2.33, P = 0.02). Moreover, subgroup meta-analyses by ethnicity were also performed to prevent the bias among different ethnic populations (Figure S1). Our subgroup meta-analyses indicated that A2M V1000I was a risk factor of AD in Italian population among 171 cases and 212 controls (OR = 1.37, 95% CI = 1.07–1.75, P = 0.01), and LPL Ser447Ter polymorphism was likely to be a protective factor of AD in Northern-American population (OR = 0.56, 95% CI = 0.35–0.91, P = 0.02, Figure S2).

## Discussion

In the present study, we carried out a systematic overview of case-control association studies for the susceptibility of AD. We screened all the available studies to harvest the eligible SNPs that were involved in at least three independent datasets. In the end, 8 SNPs of 6 AD candidate genes were included in the current meta-analyses. Our results showed significant evidence for 2 AD susceptibility SNPs (*A2M* V1000I polymorphism (OR = 1.26, 95% CI = 1.07–1.49, P = 0.007), *ABCA2* rs908832 polymorphism (OR = 1.55, 95% CI = 1.12–2.16, P = 0.009). We also observed a moderate association of AD for *CHAT* 2384G >A polymorphism (OR = 1.22, 95% CI = 1.00–1.49, P = 0.05), and an association of AD for *LPL* Ser447Ter polymorphism in the Northern-American population (OR = 0.56, 95% CI = 0.35–0.91, P = 0.02). No significant associations were found between the rest 4 SNPs and AD.

Large ethnic differences were observed for some of SNPs such as V1000I (20.8% in Germans versus 4.6% in Chinese), and Val158Met (12.4% in Colombians versus 34.8% in British). Under a moderate risk of AD (OR = 1.2), power analysis showed that there might be a lack of power for the meta-analyses of the 4 SNPs, including 5bp I/D of *A2M* (59.6%), Ser447Ter (56.6%) of *LPL*, rs908832 of *ABCA2* (31.6%) and 267C >T of *HTR6* (29.9%). These might partly explain our failure to observe significant results for the meta-analyses of most polymorphisms.

A2M is the one of the key ligands for low density lipoprotein receptor-related protein (LRP) which modulates the critical step for the clearance of A-beta, the major component of beta-amyloid [60]. A2M may regulate AD progression through its ability to mediate the degradation of A-beta [61]. V1000I polymorphism is located near the C-terminal region of A2M which inhibits the β-sheet formation and fibril-formation activities of beta-amyloid [15]. V1000I polymorphism has been shown to increase beta-amyloid directly [34]. Moreover, our meta-analysis has confirmed that *A2M* V1000I polymorphism is associated with a 26% increase in the risk of AD (P = 0.007), although validation of this finding is warranted among other ethnic populations.

As a member in the Sub-family A, ABCA2 may regulate cholesterol homeostasis and LDLR metabolism in neuronal cells [62]–[64]. ABCA2 expression has been shown to increase endogenous expression of amyloid precursor protein (APP) and the production of Aβ fragment that is a key player in AD progression [65]. SNP rs908832 is C-T polymorphism in exon 14 of the *ABCA2* gene. Our result showed a significant contribution of *ABCA2* rs908832 polymorphism to the susceptibility of AD (OR = 1.55, 95% CI = 1.12–2.16, P = 0.009). Future research is needed to clarify the mechanistic details of this polymorphism.

As the enzyme responsible for the biosynthesis of acetylcholine [66], CHAT protein is a marker of evaluating the function of basal forebrain cholinergic cells [67], the dementia severity in Alzheimer's disease [68], [69] and the density of senile plaques [70]. By modulating levels of acetylcholine, CHAT influences a wide range of cholinergic-dependent neurophysiological functions including cognitive ability [44], [71]. In early stage of AD, reduction in CHAT activity is a more sensitive indicator than the loss of cholinergic neurons in AD brains [72]. In the current study, two SNPs of *CHAT* gene were analyzed, but only 1882G >A was shown a moderate association with AD (OR = 1.22, 95% CI = 1.00–1.49, P = 0.05).

Encoding lipoprotein lipase, LPL functions as an Aβ-binding protein promoting cellular uptake and subsequent degradation of Aβ [73]. Lipoprotein lipase genes such as *APOE-ε* and *LPL,* are known to be involved in AD pathogenesis [74] [75]. LPL has a neuroprotective effect on AD by participating in the pathophysiological effects of oxidative stress [76]. Our meta-analysis indicates that *LPL* Ser447Ter polymorphism is a protective factor of AD in the Northern-American population (OR = 0.56, 95% CI = 0.35–0.91, P = 0.02) and thus supports this above speculation.

There were several limitations in our meta-analyses. Firstly, for some SNPs such as Ser447Ter of *LPL* gene, the involved samples were only limited in a few populations. The results of our meta-analyses may not stand for all ethnic populations. Future investigations in other populations are needed to clarify the contribution of the SNPs of interest to AD susceptibility. Secondly, we didn't probe the interaction of the two positive SNPs (*A2M* V1000I and *ABCA2* rs908832) and two less significant SNPs (*CHAT* 2384G >A and *LPL* Ser447Ter) with *APOE-ε4* genotype which is the strongest risk factor of AD. Thus, we can't exclude the possibility that our findings are dependent on *APOE-ε4* genotype. Thirdly, according to the disease onset age, there are two subtypes of AD, early-onset of AD (EOAD) and late-onset of AD (LOAD). A potential stratification by age may exist in the current meta-analyses, although no significant heterogeneity was found for all the 8 meta-analyses (Table S2 and S3). Among all the studies, we are able to get only three study mentioning the age of onset *A2M* 5bp I/D polymorphism. There are a total 111 EOAD and 235 LOAD cases and 129 controls younger than 65 and 338 controls with age equal to or over 65. The subgroup analysis has shown that *A2M* 5bp I/D polymorphism is not associated with AD in both young (Figure S2, OR = 0.78, 95% CI = 0.28–2.16, *P = 0.64*) and old (OR = 1.41, 95% CI = 0.62–3.18, *P = 0.41*) subgroups. As shown in the funnel plot of Figure S2, no obvious publication bias is shown for the above two meta-analyses. Fourthly, the incidence of female is higher than male in clinical and over half of the subjects participating in all the studies are female [1]. As an important factor of AD, gender should be considered as a stratifying variable for the further study exploring the diversity among the results of different studies. Due to a paucity of gender-related information, we are unable to perform the subgroup meta-analyses by gender. Fifthly, as shown in the Table S4, we have performed a thorough scanning for the criteria used to determine AD diagnosis. Among all the studies, only one study by Kan did the subgroup analysis of clinic and pathology. Due to a lack of informative subgroup analysis in the involved studies, we discontinue the subgroup analysis by the diagnosis criteria. Sixthly, as shown in the Table S5, there are inconsistencies in the presentation of the score of Mini Mental State Examination (MMSE) for controls. This may lead to the discrepancy in the association studies worldwide. Lastly but not least, we didn't include the genomewide association studies into our meta-analyses. There are a total of 33 GWA studies on AD in the GWAS catalog (http://www.genome.gov/page.cfm?pageid=26525384#searchForm) and 14 studies in the NCBI dbGap dataset (http://www.ncbi.nlm.nih.gov/gap/?term=alzheimer). All the loci in our meta-analyses are not presented among the strongest loci in those GWA studies.

In conclusion, we identified significant associations between 2 SNPs (*A2M* V1000I and *ABCA2* rs908832) and AD. Meta-analysis among 1235 cases and 1550 controls has confirmed that *A2M* V1000I is a risk factor of AD in German, Korean, Chinese, Spanish, Italian and Polish populations. Further, meta-analysis among 2234 cases and 2928 controls has confirmed that rs908832 of *ABCA2* gene is a risk factor of AD in French, American, Swiss, Greek and Japanese populations. In addition, meta-analysis among 222 cases and 259 controls indicates a moderate association of *CHAT* 2384G >A with AD in British and Korean populations. Another meta-analysis among 1112 cases and 1043 controls indicates that *LPL* Ser447Ter polymorphism is likely to be associated with a reduced risk of AD in the Northern-American population (OR = 0.56, 95% CI = 0.35–0.91, P = 0.02).

## Supporting Information

Figure S1 **Subgroup analysis by ethnicity between SNPs (5bp I/D, V1001I, rs908832, Ser447Ter).**
(TIF)

Figure S2 **Subgroup analysis by mean age of AD patient.**
(TIF)

Table S1 **The meta-analysis results of excluding each study in turn.**
(DOC)

Table S2 **The stratifying variables of the enrolled SNPs (5bp I/D, V1001I, and rs908832).**
(DOC)

Table S3 **The stratifying variables of the enrolled SNPs (1882G >A, 2384G >A, Val158Met, 267C>T, and Ser447Ter).**
(DOC)

Table S4 **Subgroup analysis by AD diagnosis criteria.**
(DOC)

Table S5 **Subgroup analysis by Mini Mental State Examination (MMSE) in control population.**
(DOC)

Checklist S1 **PRISMA Checklist.**
(DOC)
