# Supplementary figures and images for: Meta-Analyses of 8 Polymorphisms Associated with the Risk of the Alzheimer’s Disease
Source: PLoS One. 2013 Sep 10;8(9):e73129. doi: 10.1371/journal.pone.0073129 (PMC3769354; doi:10.1371/journal.pone.0073129)

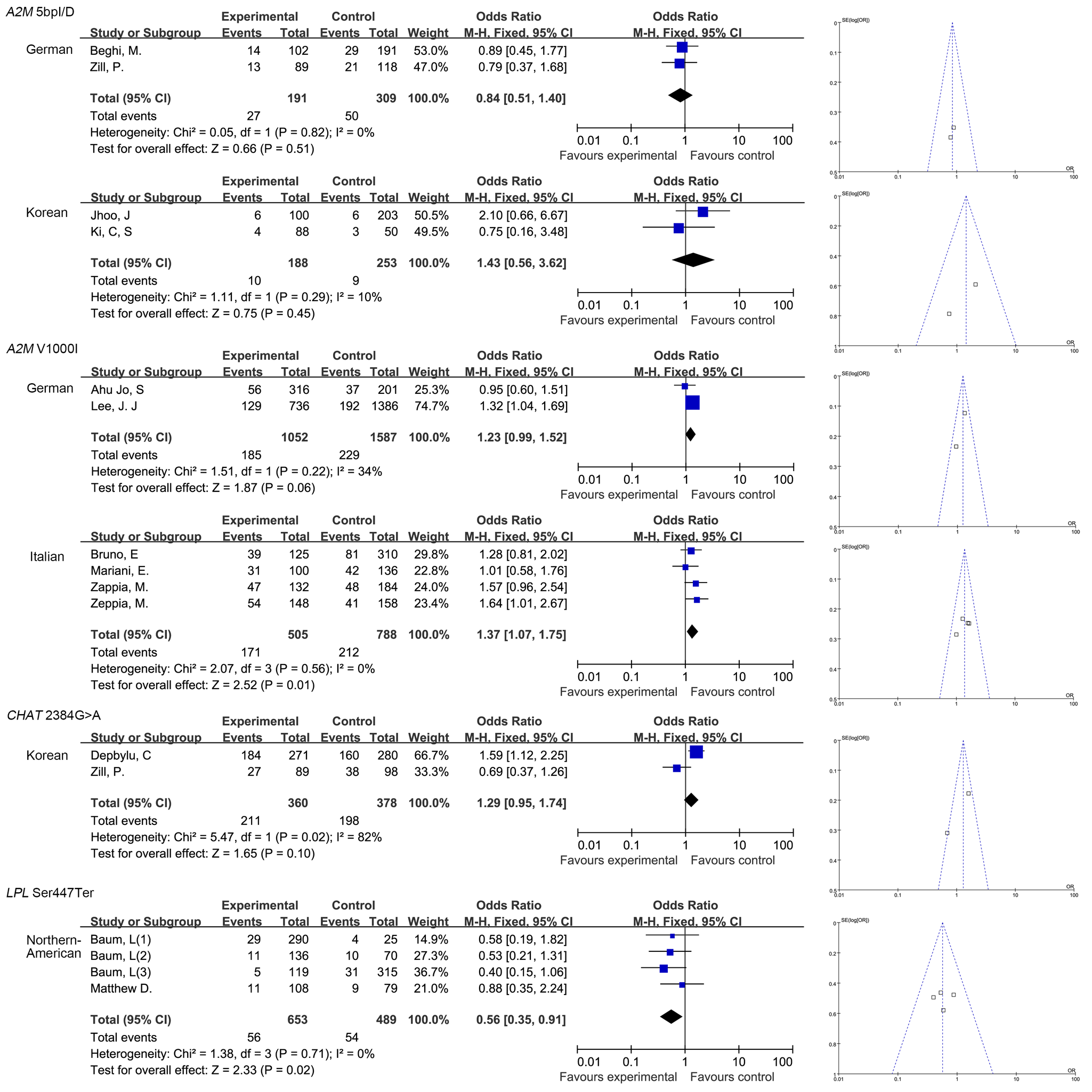

Supplement: Figure S1 — Subgroup analysis by ethnicity between SNPs (5bp I/D, V1001I, rs908832, Ser447Ter). (TIF) [file pone.0073129.s001.tif]

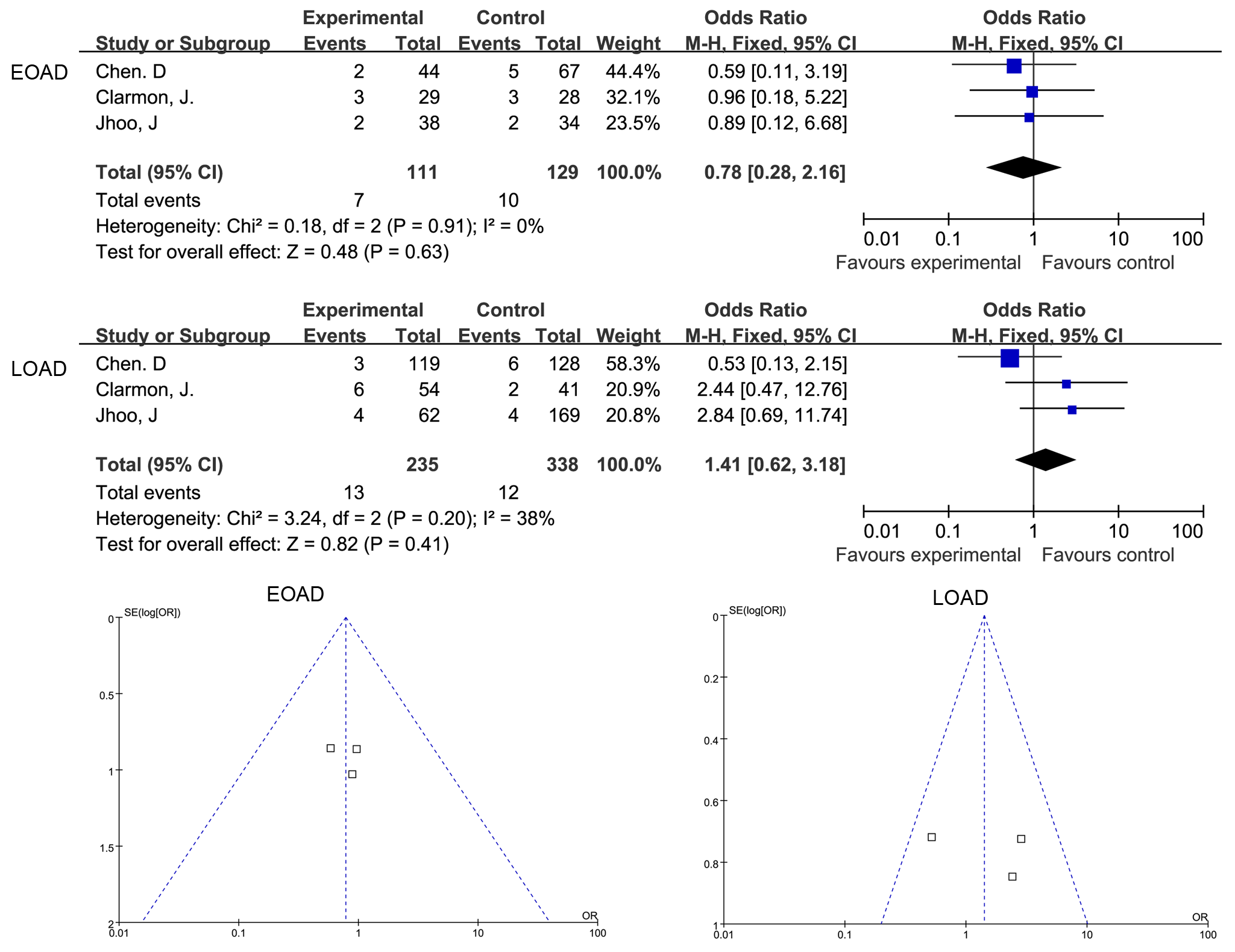

Supplement: Figure S2 — Subgroup analysis by mean age of AD patient. (TIF) [file pone.0073129.s002.tif]
