# Supplementary material for: Meta-Analyses of 8 Polymorphisms Associated with the Risk of the Alzheimer’s Disease
Source: PLoS One. 2013 Sep 10;8(9):e73129. doi: 10.1371/journal.pone.0073129 (PMC3769354; doi:10.1371/journal.pone.0073129)
Supplement: Table S1 — The meta-analysis results of excluding each study in turn. (DOC) [file pone.0073129.s003.doc]

Supplementary Table 1: The meta-analysis results of excluding each study in turn.

| **Gene** | **SNP** |  | **Study excluded from meta-analysis** | | | | | | | | |
| --- | --- | --- | --- | --- | --- | --- | --- | --- | --- | --- | --- |
|  |  |  | **# 1** | **# 2** | **# 3** | **# 4** | **# 5** | **# 6** | **# 7** | **# 8** | **# 9** |
| *A2M* | 5bpI/D | Z | 1.16 | 1.29 | 0.43 | 1.38 | 1.5 | 1.16 | 1.66 | 0.53 | 1.06 |
|  |  | P | 0.24 | 0.2 | 0.66 | 0.17 | 0.13 | 0.25 | 0.1 | 0.6 | 0.29 |
|  | V1000I | Z | 2.49 | 2.5 | 2.72 | 1.64 | 2.83 | 3.14 | 2.21 | 2.15 | 3.17 |
|  |  | P | 0.01 | 0.01 | 0.006 | 0.1 | 0.005 | 0.002 | 0.03 | 0.03 | 0.002 |
| *ABCA2* | rs908832 | Z | 1.23 | 2.79 | 2.23 | 2.82 | 2.61 | N.A. | N.A. | N.A. | N.A. |
|  |  | P | 0.22 | 0.005 | 0.03 | 0.005 | 0.009 | N.A. | N.A. | N.A. | N.A. |
| *CHAT* | 1882G>A | Z | 1.32 | 0.71 | 0.68 | N.A. | N.A. | N.A. | N.A. | N.A. | N.A. |
|  |  | P | 0.19 | 0.48 | 0.5 | N.A. | N.A. | N.A. | N.A. | N.A. | N.A. |
|  | 2384G>A | Z | 2.25 | 1.87 | 0.16 | N.A. | N.A. | N.A. | N.A. | N.A. | N.A. |
|  |  | P | 0.02 | 0.06 | 0.87 | N.A. | N.A. | N.A. | N.A. | N.A. | N.A. |
| *COMT* | Val158Met | Z | 1.09 | 0.64 | 0.48 | 0.99 | N.A. | N.A. | N.A. | N.A. | N.A. |
|  |  | P | 0.28 | 0.52 | 0.63 | 0.32 | N.A. | N.A. | N.A. | N.A. | N.A. |
| *HTR6* | 267C>T | Z | 0.43 | 0.7 | 0.16 | 0.09 | N.A. | N.A. | N.A. | N.A. | N.A. |
|  |  | P | 0.06 | 0.48 | 0.87 | 0.93 | N.A. | N.A. | N.A. | N.A. | N.A. |
| *LPL* | Ser447Ter | Z | 1.25 | 1.04 | 0.72 | 2.33 | 1.42 | N.A. | N.A. | N.A. | N.A. |
|  |  | P | 0.21 | 0.3 | 0.47 | 0.02 | 0.16 | N.A. | N.A. | N.A. | N.A. |

a: N.A. stands for not applicable. The sequences of the excluded studies follow the ones in the Tables 1 and 2.
