# Supplementary material for: Meta-Analyses of 8 Polymorphisms Associated with the Risk of the Alzheimer’s Disease
Source: PLoS One. 2013 Sep 10;8(9):e73129. doi: 10.1371/journal.pone.0073129 (PMC3769354; doi:10.1371/journal.pone.0073129)
Supplement: Table S2 — The stratifying variables of the enrolled SNPs (5bp I/D, V1001I, and rs908832). (DOC) [file pone.0073129.s004.doc]

Supplementary table 2: The stratifying variables of the enrolled SNPs (5bp I/D, V1001I, and rs908832).

| Gene | SNP | Author | Ethnic group | Gender(M/F) | Mean age | AD diagnosis criteria | MMSE | Power 1 | Power 2 |
| --- | --- | --- | --- | --- | --- | --- | --- | --- | --- |
| *A2M* | 5bpI/D |  |  |  |  |  |  | 0.77 |  |
|  |  | Zill, P. | German | 93/113 | 73.0±9.0 | NINCDS-ADRDA, DSM IV | N.A. | 0.146 | 0.085 |
|  |  | Jhoo, J. | Koreans | 66/237 | 71.2±7.8 | CERAD, DSM IV | N.A. | 0.082 | 0.053 |
|  |  | Ki, C, S. | Koreans | N.A. | N.A. | NINCDS-ADRDA | N.A. | 0.062 | 0.062 |
|  |  | Chen. D. | Chinese | 182/171 | 69.4±9.4 | NINCDS-ADRDA, MRI | ≥27 | 0.2 | 0.105 |
|  |  | Camelo, D. | Colombia | 52/110 | 69.0±8.9 | NINCDS-ADRDA | ≥26 | 0.046 | 0.063 |
|  |  | Clarmon, J. | Spanish | 72/129 | 76.7±5.4 | NINCDS-ADRDA | 26.5±2.5 | 0.128 | 0.083 |
|  |  | Zappia, M. | Italian | 136/180 | 72.3±6.4 | NINCDS-ADRDA | 25.9±3.3 | 0.229 | 0.119 |
|  |  | Prince, J. | Swedish | 157/233 | N.A. | NINCDS-ADRDA, CERAD | ≥28 | 0.2 | 0.11 |
|  |  | Beghi, M. | German | 136/157 | 74.4±10.3 | NINCDS-ADRDA | N.A. | 0.167 | 0.088 |
|  | V1000I |  |  |  |  |  |  | 0.977 |  |
|  |  | Zill, P. | German | 93/113 | 73.0 ± 9.0 | NINCDS-ADRDA, DSM IV | N.A. | 0.185 | 0.195 |
|  |  | Chen, D. | Chinese | 74/84 | 69.4±9.4 | NINCDS-ADRDA, MRI | ≥27 | 0.128 | 0.134 |
|  |  | Styczynska, M. | Polish | 76/124 | 76.4±6.4 | NINCDS-ADRDA | N.A. | 0.196 | 0.207 |
|  |  | Zappia, M. | Italian | 131/175 | 71.3±7.4 | NINCDS-ADRDA | 25.0±3.4 | 0.235 | 0.249 |
|  |  | Depbylu, C. | German | 364/187 | 70.7±9.6 | NINCDS-ADRDA | N.A. | 0.441 | 0.467 |
|  |  | Mariani, E. | Italian | 62/174 | 76.1±7.1 | NINCDS-ADRDA | ≥28 | 0.205 | 0.216 |
|  |  | Bruno, E. | Italian | N.A. | N.A. | NINCDS-ADRDA | N.A. | 0.276 | 0.293 |
|  |  | Clarmon, J. | Spanish | 72/129 | 76.7±5.4 | NINCDS-ADRDA | 26.5±2.5 | 0.183 | 0.193 |
|  |  | Zappia, M. | Italian | 136/180 | 72.3±6.4 | NINCDS-ADRDA | 25.9±3.3 | 0.24 | 0.254 |
| *ABCA2* | rs908832 |  |  |  |  |  |  | 0.444 |  |
|  |  | Mace, S. | French | 404/555 | N.A. | NINCDS-ADRDA | ≥27 | 0.155 | 0.484 |
|  |  | Minster, R. | American | N.A. | N.A. | N.A. | N.A. | 0.295 | 0.823 |
|  |  | Wollmer, M. | Swiss | N.A. | N.A. | NINCDS-ADRDA, CERAD | N.A. | 0.06 | 0.083 |
|  |  |  | Greek | N.A. | N.A. | NINCDS-ADRDA, CERAD | N.A. | 0.073 | 0.172 |
|  |  |  | Japanese | N.A. | N.A. | NINCDS-ADRDA, CERAD | N.A. | N.A. | N.A. |

a: Mean age: the mean age of AD patients was showed in the table; MMSE: Mini Mental State Examination, the MMSE was collected in control population; Power 1: The statistical power detected an OR of 1.25 at a significance level of 0.05; Power 2: the average allelic OR reported for all the associative genetic studies on AD. N.A. denotes not available.
