# Supplementary material for: Meta-Analyses of 8 Polymorphisms Associated with the Risk of the Alzheimer’s Disease
Source: PLoS One. 2013 Sep 10;8(9):e73129. doi: 10.1371/journal.pone.0073129 (PMC3769354; doi:10.1371/journal.pone.0073129)
Supplement: Table S3 — The stratifying variables of the enrolled SNPs (1882G >A, 2384G >A, Val158Met, 267C>T, and Ser447Ter). (DOC) [file pone.0073129.s005.doc]

Supplementary table 3: The stratifying variables of the enrolled SNPs (1882G>A, 2384G>A, Val158Met, 267C>T, and Ser447Ter)

| Gene | SNP | Author | Ethnic group | Gender(M/F) | Mean age | AD diagnosis criteria | MMSE | Power 1 | Power 2 |
| --- | --- | --- | --- | --- | --- | --- | --- | --- | --- |
| *CHAT* | 1882G>A |  |  |  |  |  |  | 0.891 |  |
|  |  | Harold, D. | Englishman | N.A. | 72.5±6.5 | NINCDS-ADRDA | ≥28 | 0.162 | 0.124 |
|  |  | Ozturk, A. | American | 333/668 | 76.6±5.7 | NINCDS-ADRDA | N.A. | 0.828 | 0.643 |
|  |  | Grunblatt, E. | Italian | N.A. | N.A. | NINCDS-ADRDA， MRI | >28 | 0.107 | 0.075 |
|  | 2384G>A |  |  |  |  |  |  | 0.875 |  |
|  |  | Harold, D. | Englishman | N.A. | 72.5±6.5 | NINCDS-ADRDA | ≥28 | 0.196 | 0.164 |
|  |  | Ahu Jo, S. | Korean | 186/394 | 73.0±8.0 | NINCDS-ADRDA, CERAD | N.A. | 0.283 | 0.233 |
|  |  | Lee, J. J. | Korean | 687/1435 | 73.8±9.4 | NINCDS-ADRDA | 25.3±3.2 | 0.706 | 0.603 |
| *COMT* | Val158Met |  |  |  |  |  |  | 0.948 |  |
|  |  | Forero, D. A. | Colombian | 85/185 | 73.3±8.8 | NINCDS-ADRDA | ≥27 | 0.231 | 0.076 |
|  |  | Martmez, M. F. | Spanish | 201/397 | 75.0±7.8 | DSM IV, NINCDS-ADRDA | 28.2±1.7 | 0.477 | 0.114 |
|  |  | Lanni, C. | Italian | 196/328 | 77.0±8.0 | DSM IV, NINCDS-ADRDA | 28.0±2.0 | 0.436 | 0.107 |
|  |  | Thornton, V. | Englishman | 330/366 | 75.8±0.7 | DSM IV, NINCDS-ADRDA | 28.5±0.1 | 0.542 | 0.125 |
| *HTR6* | 267C>T |  |  |  |  |  |  | 0.43 |  |
|  |  | Thome, J. | German | N.A. | N.A. | ICD-10, DSM-IV, NINCDS-ADRDA | N.A. | 0.143 | 0.05 |
|  |  | Alvarez, M. | Basque | N.A. | 70.0±7.3 | DSM-IV, NINCDS-ADRDA | N.A. | 0.128 | 0.052 |
|  |  | Kan, R. | Chinese | N.A. | 79.2±6.3 | DSM-III, NINCDS-ADRDA | N.A. | 0.135 | 0.051 |
|  |  | Orlacchio, A. | Italian | N.A. | N.A. | N.A. | N.A. | 0.135 | 0.052 |
| *LPL* | Ser447Ter |  |  |  |  |  |  | 0.746 |  |
|  |  | Fidani, L. | Caucasian | 319/694 | N.A. | NINCDS-ADRDA | N.A. | 0.362 | 0.393 |
|  |  | Baum, L. | American | N.A. | N.A. | NINCDS-ADRDA | N.A. | 0.069 | 0.118 |
|  |  |  | Canadian |  |  |  |  | 0.11 | 0.14 |
|  |  | Matthew D. | American | N.A. | N.A. | N.A. | N.A. | 0.104 | 0.122 |
|  |  | Baum, L. | European-Americans | N.A. | 75.0±6.6 | NINCDS-ADRDA | N.A. | 0.164 | 0.145 |

a: Mean age: the mean age of AD patients was showed in the table; MMSE: Mini Mental State Examination, the MMSE was collected in control population; Power 1: The statistical power detected an OR of 1.25 at a significance level of 0.05; Power 2: the average allelic OR reported for all the associative genetic studies on AD; N.A. denotes not available.
