# Supplementary material for: Meta-Analyses of 8 Polymorphisms Associated with the Risk of the Alzheimer’s Disease
Source: PLoS One. 2013 Sep 10;8(9):e73129. doi: 10.1371/journal.pone.0073129 (PMC3769354; doi:10.1371/journal.pone.0073129)
Supplement: Table S4 — Subgroup analysis by AD diagnosis criteria. (DOC) [file pone.0073129.s006.doc]

Supplementary Table 4: Subgroup analysis by AD diagnosis criteria

| **Gene** | **SNP** | **Ethnic group** | **AD diagnosis criteria** |  | **Gene** | **SNP** | **Ethnic group** | **AD diagnosis criteria** |
| --- | --- | --- | --- | --- | --- | --- | --- | --- |
| *A2M* | 5bpI/D |  |  |  | *CHAT* | 1882G>A |  |  |
|  |  | German | NINCDS-ADRDA, DSM IV |  |  |  | British | NINCDS-ADRDA |
|  |  | Korean | CERAD, DSM IV |  |  |  | American | NINCDS-ADRDA |
|  |  | Korean | NINCDS-ADRDA |  |  |  | Italian | NINCDS-ADRDA， MRI |
|  |  | Chinese | NINCDS-ADRDA, MRI |  |  | 2384G>A |  |  |
|  |  | Colombia | NINCDS-ADRDA |  |  |  | British | NINCDS-ADRDA |
|  |  | Spanish | NINCDS-ADRDA |  |  |  | Korean | NINCDS-ADRDA, CERAD |
|  |  | Italian | NINCDS-ADRDA |  |  |  | Korean | NINCDS-ADRDA |
|  |  | Swedish | NINCDS-ADRDA, CERAD |  |  |  |  |  |
|  |  | German | NINCDS-ADRDA |  | *COMT* | Val158Met |  |  |
|  | V1000I |  |  |  |  |  | Colombian | NINCDS-ADRDA |
|  |  | German | NINCDS-ADRDA, DSM IV |  |  |  | Spanish | DSM IV, NINCDS-ADRDA |
|  |  | Chinese | NINCDS-ADRDA, MRI |  |  |  | Italian | DSM IV, NINCDS-ADRDA |
|  |  | Polish | NINCDS-ADRDA |  |  |  | British | DSM IV, NINCDS-ADRDA |
|  |  | Italian | NINCDS-ADRDA |  |  |  |  |  |
|  |  | German | NINCDS-ADRDA |  | *HTR6* | 267C>T |  |  |
|  |  | Italian | NINCDS-ADRDA |  |  |  | German | ICD-10, DSM-IV, NINCDS-ADRDA |
|  |  | Italian | NINCDS-ADRDA |  |  |  | Basque | DSM-IV, NINCDS-ADRDA |
|  |  | Spanish | NINCDS-ADRDA |  |  |  | Chinese | DSM-III, NINCDS-ADRDA |
|  |  | Italian | NINCDS-ADRDA |  |  |  | Italian | N.A. |
|  |  |  |  |  |  |  |  |  |
| *ABCA2* | rs908832 |  |  |  | *LPL* | Ser447Ter |  |  |
|  |  | French | NINCDS-ADRDA |  |  |  | Caucasian | NINCDS-ADRDA |
|  |  | American | N.A. |  |  |  | American | NINCDS-ADRDA |
|  |  | Swiss | NINCDS-ADRDA, CERAD |  |  |  | Canadian | NINCDS-ADRDA |
|  |  | Greek | NINCDS-ADRDA, CERAD |  |  |  | American | N.A. |
|  |  | Japanese | NINCDS-ADRDA, CERAD |  |  |  | European-Americans | NINCDS-ADRDA |

a: NINCDS-ADRDA refers to The NINCDS-ADRDA Alzheimer's Criteria were used in the diagnosis of AD; DSM is the Diagnostic and Statistical Manual of Mental Disorders; MRI is the abbreviation of Magnetic resonance imaging; ICD-10 is the 10th revision of the International Statistical Classification of Diseases and Related Health Problems (ICD); CERAD is the Consortium to Establish a Registry for AD; N.A. denotes not available.
