# Supplementary material for: Meta-Analyses of 8 Polymorphisms Associated with the Risk of the Alzheimer’s Disease
Source: PLoS One. 2013 Sep 10;8(9):e73129. doi: 10.1371/journal.pone.0073129 (PMC3769354; doi:10.1371/journal.pone.0073129)
Supplement: Table S5 — Subgroup analysis by Mini Mental State Examination (MMSE) in control population. (DOC) [file pone.0073129.s007.doc]

Supplementary Table 5: Subgroup analysis by Mini Mental State Examination (MMSE) in control population

| **Gene** | **SNP** | **Ethnic group** | **MMSE** |  | **Gene** | **SNP** | **Ethnic group** | **MMSE** |
| --- | --- | --- | --- | --- | --- | --- | --- | --- |
| *A2M* | 5bpI/D |  |  |  | *CHAT* | 1882G>A |  |  |
|  |  | German | N.A. |  |  |  | British | ≥28 |
|  |  | Korean | N.A. |  |  |  | American | N.A. |
|  |  | Korean | N.A. |  |  |  | Italian | >28 |
|  |  | Chinese | ≥27 |  |  | 2384G>A |  |  |
|  |  | Colombia | ≥26 |  |  |  | British | ≥28 |
|  |  | Spanish | 26.5±2.5 |  |  |  | Korean | N.A. |
|  |  | Italian | 25.9±3.3 |  |  |  | Korean | 25.3±3.2 |
|  |  | Swedish | ≥28 |  |  |  |  |  |
|  |  | German | N.A. |  | *COMT* | Val158Met |  |  |
|  | V1000I |  |  |  |  |  | Colombian | ≥27 |
|  |  | German | N.A. |  |  |  | Spanish | 28.2±1.7 |
|  |  | Chinese | ≥27 |  |  |  | Italian | 28.0±2.0 |
|  |  | Polish | N.A. |  |  |  | British | 28.4±0.1 |
|  |  | Italian | 25.0±3.4 |  |  |  |  |  |
|  |  | German | N.A. |  | *HTR6* | 267C>T |  |  |
|  |  | Italian | ≥28 |  |  |  | German | N.A. |
|  |  | Italian | N.A. |  |  |  | Basque | N.A. |
|  |  | Spanish | 26.5±2.5 |  |  |  | Chinese | N.A. |
|  |  | Italian | 25.9±3.3 |  |  |  | Italian | N.A. |
|  |  |  |  |  |  |  |  |  |
| *ABCA2* | rs908832 |  |  |  | *LPL* | Ser447Ter |  |  |
|  |  | French | ≥27 |  |  |  | Caucasian | N.A. |
|  |  | American | N.A. |  |  |  | American | N.A. |
|  |  | Swiss | N.A. |  |  |  | Canadian | N.A. |
|  |  | Greek | N.A. |  |  |  | American | N.A. |
|  |  | Japanese | N.A. |  |  |  | European-American | N.A. |

a: N.A. denotes not available.
