# Supplementary material for: Meta-Analyses of 8 Polymorphisms Associated with the Risk of the Alzheimer’s Disease
Source: PLoS One. 2013 Sep 10;8(9):e73129. doi: 10.1371/journal.pone.0073129 (PMC3769354; doi:10.1371/journal.pone.0073129)
Supplement: Checklist S1 — PRISMA Checklist. (DOC) [file pone.0073129.s008.doc]

| **Section/topic** | **#** | **Checklist item** | **Reported on page #** |
| --- | --- | --- | --- |
| **TITLE** | | |  |
| Title | 1 | A meta-analysis of 8 polymorphisms associated with the risk of the Alzheimer’s disease | 1 |
| **ABSTRACT** | | |  |
| Structured summary | 2 | Aims: The aim of this study was to evaluate the combined contribution of 8 polymorphisms to the risk of Alzheimer’s disease (AD).  Methods: Through a comprehensive literature search for genetic variants involved in the AD association study, we harvested a total of 6 genes (8 polymorphisms) for the current meta-analyses. These genes consisted of A2M (5bp I/D and V1000I), ABCA2 (rs908832), CHAT (1882G>A, 2384G>A), COMT (Val158Met), HTR6 (267C>T) and LPL (Ser447Ter).  Result(s): A total of 33 studies among 9,269 cases and 10,264 controls were retrieved for the meta-analyses of 8 genetic variants. Meta-analysis showed a significant association between the A2M V1000I polymorphism and AD (odd ratio (OR) = 1.26, 95% confidence interval (CI) = 1.07-1.49, P = 0.007). Meta-analysis revealed an association of rs90883-2 allele of ABCA2 gene with an increased risk of AD (OR = 1.55, 95% CI = 1.12-2.16, P = 0.009). Meta-analysis revealed a moderate association of 2384G>A of CHAT gene with the risk of AD (OR = 1.22, 95% CI = 1.00-1.49, P = 0.05). Other meta-analyses of the rest 6 polymorphisms suggested a lack of association with the risk of AD.  Conclusions: Our results confirmed that A2M V1000I polymorphism was associated with susceptibility to AD in German, Korean, Chinese, Spanish, Italian and Polish populations. A significant association was found between rs90883 of ABCA2 gene and susceptibility to AD in French, American, Swiss, Greek and Japanese populations. And 2384G>A of CHAT gene was related to the risk of AD in [British](dict://key.0895DFE8DB67F9409DB285590D870EDD/Englishmen) and Korean populations. | 2 |
| **INTRODUCTION** | | |  |
| Rationale | 3 | Alzheimer's disease (AD) is the most common form of dementia among people over 65 years of age. AD is predicted to affect 1 in 85 people globally by 2050. As an incurable degenerative disease, AD gets worse gradually and eventually leads to death. The features of AD development consist of loss of cognitive functions such as thinking, remembering, and reasoning, and ultimately cause death. The averaged life expectancy after AD diagnosis is seven years. Although hundreds of clinical trials have been conducted to find ways to treat the disease, none has claimed its effect of stopping or reversing the progressive symptoms. Because AD patients rely on others for assistance, it has imposed great economic costs on society. | 3 |
| Objectives | 4 | In the present study, we aim to evaluate the combined contribution of the SNPs in these genes to AD susceptibility in different populations using a meta-analysis approach. | 4 |
| **METHODS** | | |  |
| Protocol and registration | 5 | No |  |
| Eligibility criteria | 6 | The criteria for the selection of literatures in the meta-analyses were as followed: (1) the study was case-control association study; (2) allele or genotype information is available; (3) the involved genetic variants have not been studied in previous meta-analysis. | 5 |
| Information sources | 7 | Reports published from 1999 to 2012 were included. | 5 |
| Search | 8 | Literatures were searched through the online databases from 1999 to 2012 using the following key words: “alzheimer’s disease, association, SNP or polymorphism or variant or variation or mutation”. The involved databases include PubMed, Chinese National Knowledge infrastructure (CNKI), Embase, SpringerLink, and ScienceDirect. Reference lists in the harvested literatures were looked up for additional case-control studies. | 5 |
| Study selection | 9 | After removing the duplicated publications, we harvested 3270 articles. Among them, 1417 studies were discarded for their involvement in the previous meta-analyses. For the rest 476 studies, we filtered out a total of 428 articles because they failed to accumulate at least three independent genotypic datasets for the same genetic variants. At last, there were 33 case-control studies with 8 polymorphisms for the current meta-analyses. | 5 |
| Data collection process | 10 | We collected relevant information via reading the full text. If the reports were not given the data of the allele clearly, we calculated the allele by the information of genotypes in the papers. | 5 |
| Data items | 11 | For the all SNPs (5bp I/D, V1000I, rs908832, 1882G>A, 2384G>A, Val158Met, 267C>T, rs2233678, rs2233679, Ser447Ter), we listed the number of various genotypes and alleles and the other information. | 5 |
| Risk of bias in individual studies | 12 | Heterogeneity was tested by the Cochran’s Q statistic and I2 test. A I2 < 50% denoted a non-signficant heterogeneity among the involved studies in the meta-analysis and fixed-effect model was used in the meta-analyses. | 5 |
| Summary measures | 13 | The meta-analyses were done using the Review Manager 5.0 software. Total ORs with 95% CIs were estimated to evaluate the strength of the association between polymorphisms and AD risk. | 5 |
| Synthesis of results | 14 | For the SNPs, we evaluated ORs of minor Allele in the total alleles. | 5 |

Page 1 of 2

| **Section/topic** | **#** | **Checklist item** | **Reported on page #** |
| --- | --- | --- | --- |
| Risk of bias across studies | 15 | The funnel plot was used to evaluate the publication bias in the meta-analysis. A two-sided P value < 0.05 in the Z-test was treated as significant. | 5 |
| Additional analyses | 16 | No. |  |
| **RESULTS** | | |  |
| Study selection | 17 | Our search for the case-control studies of AD retrieved 3,351 articles from PubMed, Embase, Web of Science, CNKI and Wanfang from 1999 to 2012. After removing the duplicated publications, we harvested 3270 articles. Among them, 1417 studies were discarded for their involvement in the previous meta-analyses. For the rest 476 studies, we filtered out a total of 428 articles because they failed to accumulate at least three independent genotypic datasets for the same genetic variants. At last, there were 33 case-control studies with 8 polymorphisms for the current meta-analyses (Figure 1). | 6 |
| Study characteristics | 18 | The retrieved information consisted of the first author, the year of publication, the number of participants with the different allele (patients and healthy controls), and the odds ratios (ORs) values with 95% confidence intervals (CIs). In total, 33 publications include 8 polymorphisms were included in the current meta-analyses. | 6 |
| Risk of bias within studies | 19 | The results of tests of heterogeneity are in figure 2 and figure3. | 21 |
| Results of individual studies | 20 | The results of individual studies are in table1 and table2. | 17-20 |
| Synthesis of results | 21 | The results are in table1 and table2. | 17-20 |
| Risk of bias across studies | 22 | The results of publication bias did not observed (figure4) | 21 |
| Additional analysis | 23 | No |  |
| **DISCUSSION** | | |  |
| Summary of evidence | 24 | In the present study, we carried out a systematic overview of case-control association studies for the susceptibility of AD. We screened all the available studies to harvest the eligible SNPs that were involved with at least three independent datasets. In the end, 8 SNPs of 6 AD candidate genes were included in the current meta-analyses. Our results showed significant evidence for 2 AD susceptibility SNPs (A2M V1000I polymorphism (OR = 1.26, 95% CI = 1.07-1.49, P = 0.007), ABCA2 rs908832 polymorphism (OR = 1.55, 95% CI = 1.12-2.16, P = 0.009). We also observed a moderate association of AD for CHAT 2384G>A polymorphism (OR = 1.22, 95% CI = 1.00-1.49, P = 0.05), and a boundary association of AD for LPL Ser447Ter polymorphism (OR = 0.77, 95% CI = 0.58-1.02, P = 0.07). For the rest 4 SNPs, our meta-analyses were unable to find significant associations of them with AD. | 7 |
| Limitations | 25 | There were several limitations in our meta-analyses. Firstly, for some SNPs such as Ser447Ter of LPL gene, the involved samples were only limited in a few populations. The results of our meta-analyses may not stand for all ethnic populations. Future investigations in other populations are needed to clarify the contribution of the SNPs of interest to AD susceptibility. Secondly, we didn’t probe the interaction of the two positive SNPs (A2M V1000I and ABCA2 rs908832) and two less significant SNPs (CHAT 2384G>A and LPL Ser447Ter) with APOE-ε4 genotype which is the strongest risk factor of AD. Thus, we can’t exclude the possibility that our findings are dependent on APOE-ε4 genotype. Thirdly, according to the disease onset age, there are two subtypes of AD (early-onset of AD and late-onset of AD). Among the case-control studies in the meta-analyses, we didn’t find enough information to differentiate the two subtypes among the included datasets. Therefore, a potential stratification may exist in the current meta-analyses, although no significant heterogeneity was found for all the 8 meta-analyses. | 8 |
| Conclusions | 26 | In conclusion, we identify significant associations between 2 SNPs (A2M V1000I and ABCA2 rs908832) and AD. Meta-analysis among 1235 cases and 1550 controls has confirmed that A2M V1000I gene is a risk factor of AD in German, Korean, Chinese, Spanish, Italian and Polish populations. Meta-analysis among 2234 cases and 2928 controls has confirmed that rs908832 of ABCA2 gene is a risk factor of AD in French, American, Swiss, Greek and Japanese populations. In addition, meta-analysis among 222 cases and 259 controls indicates a moderate association of CHAT 2384G>A with AD in British and Korean populations. Another meta-analysis among 1538 cases and 1138 controls indicates that LPL Ser447Ter has a trend of association with the risk of AD in American, north-American, Canadian, Caucasian and European-American populations. | 9 |
| **FUNDING** | | |  |
| Funding | 27 | The research was supported by the grants from the National Natural Science Foundation of China (31100919), K. C. Wong Magna Fund in Ningbo University, Ningbo social development research projects (2012C50032), Science and Technology Innovation team of Ningbo (2011B82014), the neurobiological mechanisms of drug-reward: role of the habenula National Natural Science Foundation of China (81171257), and Research Fund in Ningbo University (XKL11D2117) | 10 |

*From:*  Moher D, Liberati A, Tetzlaff J, Altman DG, The PRISMA Group (2009). Preferred Reporting Items for Systematic Reviews and Meta-Analyses: The PRISMA Statement. PLoS Med 6(6): e1000097. doi:10.1371/journal.pmed1000097

For more information, visit: **www.prisma-statement.org**.

Page 2 of 2
